# Defining Satisfactory Methods of Treatment in Rare Diseases When Evaluating Significant Benefit–The EU Regulator's Perspective

**DOI:** 10.3389/fmed.2021.744625

**Published:** 2021-08-27

**Authors:** Maria E. Sheean, Frauke Naumann-Winter, Giuseppe Capovilla, Maria Elisabeth Kalland, Eva Malikova, Segundo Mariz, Darius Matusevicius, Robert Nistico, Brigitte Schwarzer-Daum, Stelios Tsigkos, Kyriaki Tzogani, Kristina Larsson, Armando Magrelli, Violeta Stoyanova-Beninska

**Affiliations:** ^1^European Medicines Agency, Amsterdam, Netherlands; ^2^Committee of Orphan Medicinal Products, European Medicines Agency, Amsterdam, Netherlands; ^3^Bundesinstitut für Arzneimittel und Medizinprodukte, Bonn, Germany; ^4^Carlo Poma Hospital, Mantova, Italy; ^5^Fondazione Poliambulanza, Brescia, Italy; ^6^Statens Legemiddelverk, Oslo, Norway; ^7^State Institute for Drug Control, Bratislava, Slovakia; ^8^Department of Pharmacology and Toxicology, Comenius University, Bratislava, Slovakia; ^9^Läkemedelsverket, Uppsala, Sweden; ^10^Malta Medicines Authority, San Gwann, Malta; ^11^Medical University of Vienna, Vienna, Austria; ^12^National Center for Drug Research and Evaluation, Istituto Superiore di Sanità, Rome, Italy; ^13^College ter Beoordeling van Geneesmiddelen, Utrecht, Netherlands

**Keywords:** orphan designation, satisfactory methods of treatment, significant benefit, orphan regulation, committee for orphan medicinal products

## Abstract

Since the implementation of the EU Orphan Regulation in 2000, the Committee for Orphan Medicinal Products at the European Medicines Agency has been evaluating the benefits of proposed orphan medicines vs. satisfactory treatment methods. This type of evaluation is foreseen in the Orphan Regulation as the orphan designation criterion called the “significant benefit.” In this article, based on 20 years of experience, we provide a commentary explaining what is considered a satisfactory method of treatment in the context of the EU Orphan Regulation and for the purpose of the assessment of significant benefit. We discuss the challenges posed by continuously changing clinical practise, which is associated with the increasing number of treatment options, evolving nature of medicinal therapeutic indications and our understanding of them.

## Defining a Satisfactory Method of Treatment

According to the European Union (EU) Orphan Regulation (1), a candidate medicine can be awarded an orphan status if it fulfils a set of defined criteria. The medicine must be intended for the treatment, prevention, or diagnosis of a disease that is life-threatening or chronically debilitating. The medicine must either target a disease affecting no more than 5 in 10,000 persons in the EU or be unlikely to generate sufficient returns to justify the investment needed for its development. In addition, if a satisfactory method of diagnosis, prevention or treatment of the condition concerned exists, the medicine must be of significant benefit to those affected by the condition. The eligibility of a candidate medicine to orphan designation (OD) is assessed by a dedicated Committee for Orphan Medicinal Products (COMP) at the European Medicines Agency (EMA).

The requirement to show that a product for which an OD is applied will be of significant benefit to those affected by the orphan condition in cases where other satisfactory methods exist is a unique criterion in the EU Orphan Regulation framework. The concept of significant benefit has been discussed previously in a number of publications (2, 3). The need to demonstrate significant benefit is of particular importance as it may be effectively gatekeeping in nature (e.g., blocking new products from obtaining a designation or preventing incentives such as the 10 years of market exclusivity due to lack of adequate comparative data).

Therefore, to provide further information, in this article we aim to explain what constitutes a satisfactory method of treatment. This naturally depends on the specific rare disease the medicinal product intends to diagnose, prevent, or treat. The evaluation needs to be performed both at the initial stage of OD and when reviewing maintenance of orphan status at the time of marketing authorisation (MA). The assessment at the time of OD is made early in the medicine development, often at the stage of non-clinical studies (4, 5), whereas the assessment at maintenance stage takes place after the medicine receives a positive opinion from the Committee of Human Medicinal Products (CHMP) at EMA following a positive benefit/risk assessment.

To demonstrate significant benefit, an assessment is conducted by the COMP based on data provided by the applicant and established evidence from the public domain. The assessment concerns the new product vis-a-vis relevant comparators currently used in Europe for the treatment of the proposed orphan condition. The COMP considers the standard of care in identifying appropriate comparators and the target patient population suitable for the analysis of significant benefit.

MA is granted if the benefit/risk balance is positive (6). Medicines authorised for a given indication throughout EU based on such positive benefit/risk balance are considered as satisfactory within the meaning of the Orphan Regulation (1). Medicinal products may be deemed as being authorised in the Community via either a national, decentralised or centralised procedure, hence authorised in a single, several or all member states (7, 8). The definition of a satisfactory method of treatment is based on a reference to the terms of the MA as described in the Summary of Product Characteristics (SmPC) (7, 8). Therefore, medicinal products which are authorised for the treatment of the disease as such or, at the very least, the same set of essential symptoms associated with the disease, can be considered as satisfactory methods of treatment (7, 8). A product that is administered or applied outside of the approved SmPC (used “off-label”) should not be considered a satisfactory method according to the Orphan Regulation (8). Similarly, medicines applied under hospital exemption would not be considered satisfactory (8). This is because hospital exemption is typically given under exclusive physician's responsibility for a medicine that is not used or produced routinely, and where the benefits and risks associated with such therapy are not well-known.

In some cases, a medicine may be taken into account when it is authorised for a broader patient population than the targeted orphan condition. Examples include older products such as corticosteroids or antiepileptics with broad labels and use in many diseases. In addition, medicines may have different national authorisations, and hence different indication wording in the SmPC at national level. It suffices, however, that the condition (or a set of essential symptoms) in question is mentioned in the approved SmPC in one member state for it to be considered as a satisfactory method of treatment for the purpose of an OD.

Moreover, in some disease areas, a non-pharmacological therapy can be considered a satisfactory method if there is public and widespread consensus among clinicians in the field as to the value of such treatment (e.g., surgery, radiotherapy, diet etc.). As an example, diet in the case of phenylketonuria is considered satisfactory due to its high level of efficacy in the treatment of the disease. In exceptional cases, medicines prescribed for individual patients in the hospital (commonly known as the “magistral formulas”) may be considered as satisfactory treatment if they are well-known and safe and this is a general practise in the EU (8).

The meaning of the word “satisfactory” should not be confused with similar concepts, such as “efficacious,” each word bearing a different regulatory meaning. The word “efficacious” refers to the efficacy or effectiveness of the product in a particular condition. However, the fact that a medicine is not curative or fully effective does not imply that it is not considered “satisfactory” from a regulatory point of view as long as the benefit/risk balance is deemed positive.

## Cases of Challenging Decisions and Lessons Learnt

Despite the guidance from the European Commission (EC) (7, 8), there are cases when the decision on whether a medicine can be considered satisfactory can be challenging. An example of such a “grey area” are medicines which treat well-characterised and serious symptoms of a disease (e.g., antiseizure medications (ASMs) or immunosuppressants). For example, most ASMs are authorised for treatment of specific seizure types, like e.g., focal seizures, generalised tonic-clonic seizures, myoclonic seizures, etc. However, only few medicines are approved for specific conditions where these various types of seizures typically manifest and, therefore, different ASMs are used. As such, ASMs which are treating characteristic set of symptoms of the disease could potentially be considered as a satisfactory method for the purpose of the significant benefit assessment.

The situation can also be challenging when authorised medicines for various reasons are no longer used or have just recently been approved. In the first instance, there might be change in current clinical practise making a comparison to an old and unused product irrelevant; in the latter case, the product may be “too new” to allow for a comparative analysis in the context of a significant benefit discussion. In both cases, the approved medicines must be captured in the description of the standard of care. In case of recent approval of a new medicine for treatment of the same condition, it is still expected that a new medicine shows significant benefit vs. the one recently approved (9).

## Existing Methods of Treatment

It is of great importance for the COMP to capture all existing methods of treatment across the EU. On occasion, significant differences are noted across member states regarding medicines in their licencing, terms of MA, availability and patient access. While the authorisation and specific label are to be taken into consideration, the availability and access fall outside of the COMP remit. The most complete standard of care and publicly available guidance on how authorised medicines are used are considered for the purpose of assessing an application for an OD. It should be noted that there might be a difference in the list of satisfactory methods discussed at time of initial OD and when reviewing maintenance of orphan status at time of a MA stage when the standard of care may have evolved. In order to ascertain the existence of satisfactory methods at the time of an orphan maintenance stage, there must be a full overlap of therapeutic indications and patient populations between the candidate and the authorised medicinal product(s). If the therapeutic indication covered by the candidate is broader than that of the authorised medicinal product(s), then the latter will not be considered satisfactory for the purpose of the significant benefit assessment (10).

The task of comprehending the current EU standard of care becomes increasingly more complex due to continuous addition of new authorised medicines for orphan conditions, as this leads to notable changes in how patients are managed. This applies specially to “crowded” therapeutic areas such as seen in oncology, where many treatment options exist but their clinical use in practise is not well-structured and standardised. Management of multiple myeloma can be mentioned as an example to illustrate the difficult task of developers and regulators in proper contextualisation of the therapeutic effects observed [see recent Orphan Maintenance Assessment reports on Blenrep (11) and Nexpovio (12)].

## The Aim of Maximum Transparency

It is the aim of the COMP to make publicly available documents transparent and informative, for the purpose of sharing evaluations with all stakeholders that could be interested in comparisons vs. standard of care. That said, it should be noted that the definition of satisfactory methods used by the COMP might be different compared to the standard of care considered relevant for a clinician managing individual patients or suitable for an individual HTA. These stakeholders often focus on national treatment standards or the most commonly used treatment options and may have different inclusion criteria when it comes to treatment methods.

## Conclusions

In conclusion, significant benefit is a unique European criterion which needs to be evaluated by the COMP at initial OD and at time of MA. Significant benefit is assessed based on comparative data between the new product and all existing satisfactory methods of treatment. An authorised medicine for a given condition based on a positive benefit/risk balance is considered a satisfactory method. Such medicines are easy to identify when comparing the proposed medicine to authorised medicines with similarly worded therapeutic indications. A number of special considerations have been mentioned above, which are discussed by the COMP on a case-by-case basis. There may be discrepancies between the standard of care and a set of comparators considered for significant benefit, because not all medicines in use are approved for the same indication and not all non-pharmacological methods may be included. The list of comparators may also differ at initial OD and at time of MA, because of restricted wording of the approved therapeutic indication at MA, or because more satisfactory treatments were authorised after the granting of the initial OD. If in doubt, an applicant may always inquire with the EMA to receive appropriate regulatory guidance. However, a comprehensive description of the standard of care based on treatment guidelines and a comparative discussion are always recommended. Following the spirit of the EU orphan legislation, dedicated research of all medicines for specific rare diseases is encouraged and may be rewarded with the orphan incentives whenever significant benefit has been clearly demonstrated over existing satisfactory methods of treatment.

## Author Contributions

MS and FN-W conducted research, led discussions on the topic, prepared and reviewed the manuscript. All authors participated in discussions, contributed to drafting, and reviewing of the manuscript.

## Author Disclaimer

The views expressed in this article are the personal views of the authors and may not be understood or quoted as being made on behalf of or reflecting the position of the European Medicines Agency or one of its committees or working parties.

## Conflict of Interest

The authors declare that the research was conducted in the absence of any commercial or financial relationships that could be construed as a potential conflict of interest.

## Publisher's Note

All claims expressed in this article are solely those of the authors and do not necessarily represent those of their affiliated organizations, or those of the publisher, the editors and the reviewers. Any product that may be evaluated in this article, or claim that may be made by its manufacturer, is not guaranteed or endorsed by the publisher.
